# Myelin basic protein and neurofilament H in postmortem cerebrospinal fluid as surrogate markers of fatal traumatic brain injury

**DOI:** 10.1007/s00414-021-02606-y

**Published:** 2021-04-24

**Authors:** Simone Bohnert, Christoph Wirth, Werner Schmitz, Stefanie Trella, Camelia-Maria Monoranu, Benjamin Ondruschka, Michael Bohnert

**Affiliations:** 1 Institute of Forensic Medicine, University of Wuerzburg, Versbacher Str. 3, 97078 Wuerzburg, Germany; 2 Institute of Biochemistry and Molecular Biology I, Biozentrum - Am Hubland, 97074 Wuerzburg, Germany; 3 Department of Neuropathology, Institute of Pathology, University of Wuerzburg, Josef-Schneider Str. 2, 97080 Wuerzburg, Germany; 4 Institute of Legal Medicine, University Medical Center Hamburg-Eppendorf, Butenfeld 34, 22529 Hamburg, Germany

**Keywords:** CSF, Cerebrospinal fluid, Forensic neuropathology, Forensic neurotraumatology, Biomarker, Biofluid

## Abstract

The aim of this study was to investigate if the biomarkers myelin basic protein (MBP) and neurofilament-H (NF-H) yielded informative value in forensic diagnostics when examining cadaveric cerebrospinal fluid (CSF) biochemically via an enzyme-linked immunosorbent assay (ELISA) and comparing the corresponding brain tissue in fatal traumatic brain injury (TBI) autopsy cases by immunocytochemistry versus immunohistochemistry. In 21 trauma and 19 control cases, CSF was collected semi-sterile after suboccipital puncture and brain specimens after preparation. The CSF MBP (*p* = 0.006) and NF-H (*p* = 0.0002) levels after TBI were significantly higher than those in cardiovascular controls. Immunohistochemical staining against MBP and against NF-H was performed on cortical and subcortical samples from also biochemically investigated cases (5 TBI cases/5 controls). Compared to the controls, the TBI cases showed a visually reduced staining reaction against MBP or repeatedly ruptured neurofilaments against NF-H. Immunocytochemical tests showed MBP-positive phagocytizing macrophages in CSF with a survival time of > 24 h. In addition, numerous TMEM119-positive microglia could be detected with different degrees of staining intensity in the CSF of trauma cases. As a result, we were able to document that elevated levels of MBP and NF-H in the CSF should be considered as useful *neuroinjury* biomarkers of traumatic brain injury.

**Supplementary Information:**

The online version contains supplementary material available at 10.1007/s00414-021-02606-y.

## Introduction

Traumatic brain injury (TBI), isolated or combined with other injuries, is a relevant post-traumatic prognostic factor for morbidity and mortality. In Germany, about 272,000 people suffer a TBI every year, and more than 5000 patients die as consequence [1]. With an incidence of 332/100,000 inhabitants, TBI is even more common than stroke (215/100,000 inhabitants) [2, 3]. The prognosis of patients depends on the primary mechanical brain damage as well as on the development of secondary sequelae such as intracranial pressure increase, ischemia, and hypoxia [4, 5]. To more accurately assess this primary and secondary brain damage, the clinical use of central nervous system (CNS) biomarkers has been repeatedly tested to diagnose TBI and to better understand the orchestration of secondary responses. So far, mainly structural proteins of the cell compartments of the CNS in serum and cerebrospinal fluid (CSF) have been analyzed as markers of acute brain trauma [6–10].

Investigations of fatal TBI cases have always been a classical domain of forensic medicine with regard to traumatological and biomechanical aspects as well as in the contextual assessment [11]. Currently, autopsy and histological examination of the traumatized tissue [12] are the main investigations used in the forensic postmortem routine to evaluate lethality and survival time (wound age). In addition to forensic neuropathological diagnostic methods, postmortem biochemical analyses of various cytokines, acute phase proteins, CNS biomarkers [7, 13–17], or Na^+^-glucose transporters [18] in CSF and brain tissue as well as investigations of the early tissue reaction of local microglia after trauma are meanwhile increasingly performed [19]. Furthermore, the applicability of immunocytochemical staining in postmortem CSF could be demonstrated [20].

Due to the extended length of axonal fiber tracks within the CNS, axons are particularly vulnerable to physical trauma of the brain tissue resulting in white matter damage [21]. In acute demyelination, it was demonstrated that microglia as the major cellular component of the innate immune system in the CNS preferentially accrue in and monopolize the CNS lesion site [22] in a direct and immediate immunological reaction (neuroinflammation) [23], but this tissue reaction provides no *direct* information regarding the amount of axonal injury in detail. Axonal injury commonly occurs in both focal and diffuse brain trauma due to shear forces and can be found in TBI of all severities [24]. Thus, investigating biomarkers or proteins expressed mainly or exclusively in the *axonal* parts of neurons, e.g., the myelin sheath or the axonal cytoskeleton, might help to represent the axonal component of TBI pathology and supply biochemical answers to the physical trauma.

**Fig. 1:**
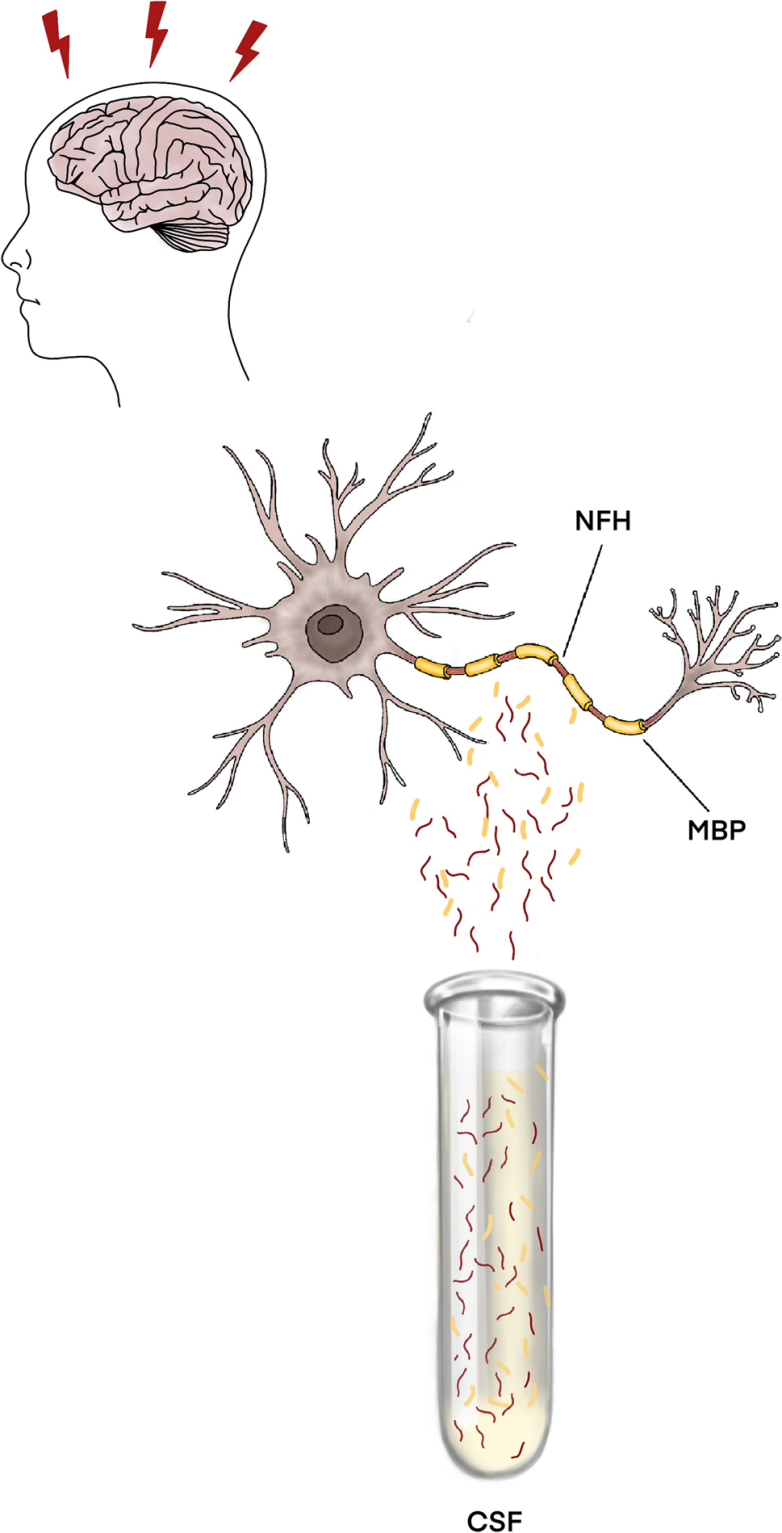
After axonal damage in the context of traumatic brain injury (TBI), axonal structural components such as parts of the myelin sheath (MBP) or the cytoskeleton (neurofilament H) enter the extracellular space and can thus be measured quantitatively in the CSF by biochemical methods, for example, an ELISA (enzyme-linked immunosorbent assay)

Apart from myelin oligodendrocyte glycoprotein (MOG), myelin basic protein (MBP) is one of the most abundant proteins (30% protein content of myelin) in the white matter [25]. It is a key structural component of the multi-layered myelin sheath covering nerve fibers. MBP maintains the correct structure of myelin, interacting with lipids in the myelin membrane [26]. In myelinated fiber tracks of the white matter, MBP degradation by proteases such as calpain results in degradation of axons and the myelin sheath (demyelination) [27, 28]. Thus, under these conditions, MBP or its fragmented or degraded forms might be released into the extracellular matrix after TBI (see Fig. 1) and thus can be measured in CSF. In human studies of adult and pediatric TBI patients, MBP was found to be elevated in serum and CSF post-traumatically during lifetime [29–34]. In the postmortem field, MBP was considered as an early marker of severe and moderate TBI in biochemical tests using CSF [35].

Neurons of the CNS contain type IV intermediate filaments, also known as neurofilaments (NFs), which are composed of an assembly of three chains: a light chain (NF-L) weighing 68 kDa, an intermediate chain (NF-M) weighing 190 kDa, and a heavy chain (NF-H) weighing 210 kDa [36]. NFs are a major cytoskeleton component and provide the structure and diameter of an axons [37]. After axonal damage, NF chains may be dissociated from the cytoskeleton and released into the cytosol or possibly the extracellular fluid, especially if the cell membrane integrity is altered. Here, NFs could serve as biomarkers of traumatic axonal injury (see Fig. 1). In serum and CSF levels of rats, NF-H was shown to correlate with the severity of a mechanical impact in an impact acceleration model [38]. Zurek et al. [39] reported the efficacy of serum NF-H measurement in predicting the injury type and outcome in children after TBI. In this study, levels of NF-H were significantly higher in patients with diffuse axonal injury (DAI) on initial CT scans compared to those without DAI [39]. Vajtr et al. [40] compared serum NF-H concentrations between DAI and focal injury showing that the median serum NF-H was higher in DAI compared to focal TBI. These findings seem to display a more specific role of NF-H in axonal injury [41], especially in distinguishing DAI from focal injury, forming the rationale for choosing NF-H as an example of different NFs in this study.

Due to the multi-component pathology in TBI, it would be ideal to define biomarkers closely matching with various pathological processes including *axonal* components of TBI. This has not yet been done in forensic pathology studies up to now.

The aim of the present study was, therefore, to biochemically investigate the potential use of MBP and NF-H as promising postmortem cerebral *neuroinjury* biomarkers for determining TBI as the cause of death compared to natural causes.

## Material and methods

### Sampling and processing

CSF samples were collected by semi-sterile puncture of the suboccipital space during head evisceration in a total of 40 forensic autopsy cases. The samples are divided into cases with lethal TBI (total number *n* = 21, case characteristics are indicated in detail in Table 1) and compared to a cohort of cardiovascular fatalities (CVF) as controls (total number *n* = 19; *n* = 7 sudden cardiac death, *n* = 9 acute myocardial infarction, *n* = 3 ruptured aortic aneurysm; sex, age, and post-mortem interval (PMI) distribution among controls in Table 2). Trauma cases were collected with different survival times ranging between hours and weeks to cover a broader time interval of survival. The cases were derived from routine medicolegal autopsies performed at the Institute of Forensic Medicine of the University of Wuerzburg. Exclusion criteria for sampling were as follows: presence of former CNS injuries (“repetitive” trauma) or neurodegenerative diseases and putrefactive tissue changes. Police and medical records were used to obtain information regarding history of older CNS injuries.

**Table 1:** Characteristics of all traumatic brain injury (TBI) and control cases investigated in this study

Case number	Sex	Age	PMI	Cause of death (underlying mechanism)
				TBI < 24 h
1	m	25	6	Intracranial hemorrhage (car accident)
2	m	42	5	Intracranial hemorrhage (car accident)
3	m	66	8	Intracranial hemorrhage (car accident)
4	f	80	5	Cortical contusion (fall)
5	m	82	2	Intracranial hemorrhage (car accident)
6	f	91	5	Cortical contusion (fall)
7	m	81	5	Intracranial hemorrhage (car accident)
8	f	87	6	Cortical contusion (fall)
9	m	55	5	Intracranial hemorrhage (car accident)
10	m	75	7	Intracranial hemorrhage (car accident)
11	m	55	5	Intracranial hemorrhage (car accident)
				TBI < 9 days
12	f	80	4	Cortical contusion (fall)
13	m	73	8	Intracranial hemorrhage (car accident)
14	f	65	2	Intracranial hemorrhage (car accident)
15	m	88	6	Cortical contusion (fall)
16	m	66	9	Intracranial hemorrhage (car accident)
				TBI ≤ 1 month
17	m	82	4	Cortical contusion (fall)
18	m	87	3	Cortical contusion (fall)
19	m	84	4	Cortical contusion (fall)
20	f	72	9	Intracranial hemorrhage (car accident)
21	f	88	6	Cortical contusion (fall)

**Table 2:** Sex, age, and post-mortem interval (PMI) distribution among controls

Case number	Sex	Age	PMI	Cause of death
				Cardidovascular fatalities
1	m	50	5	Ruptured aortic aneurysm
2	f	55	2	Ruptured aortic aneurysm
3	f	63	2	Ruptured aortic aneurysm
				Sudden cardiac death
4	m	66	4	Coronary artery disease
5	f	84	6	Aortic valve stenosis
6	f	61	5	Coronary artery disease
7	m	52	6	Coronary artery disease
8	m	55	4	Coronary artery disease
9	f	93	8	Aortic valve stenosis
10	f	21	4	Myocarditis
11	m	44	5	Acute myocardial infarction
12	m	52	8	Acute myocardial infarction
13	m	77	6	Acute myocardial infarction
14	m	57	13	Acute myocardial infarction
15	m	35	3	Acute myocardial infarction
16	f	29	1	Acute myocardial infarction
17	m	54	5	Acute myocardial infarction
18	m	33	3	Acute myocardial infarction
19	f	86	6	Acute myocardial infarction

The local Ethics Committee has approved the study (local no. 203/15).

The study included 15 females and 25 males ranging from 21 to 91 years with a PMI varying between 1 and 13 days. CSF samples were immediately centrifuged at 5000 rpm for 5 min at 4 °C, and the supernatants were aliquoted and stored at – 80 °C without any thawing cycles until analysis to allow for both cytological and biochemical analyses, respectively. CSF MBP and NF-H concentrations were measured using commercially available double-sandwich ELISA kits according to the manufacturers’ protocols (*MBP:* Mybiosource, San Diego, USA; Cat. No. MBS261463; *NF-H*: Cusabio, Houston, USA; Cat. No. CSB-E16097h). In brief, standards and CSF samples were incubated in microplate wells precoated with anti-human MBP and anti-human NF-H antibodies. Then, they were incubated with a biotin-labelled anti-human MBP/anti-human NF-H antibody solution following incubation with a streptavidin–horseradish peroxidase conjugate. The plates were washed four times with washing buffer between each step. After the last washing step, the substrate was added. The reaction was stopped by adding an acidic solution called “stop solution” in the manufacturers’ protocols after 10 min. The absorbance of the resulting color product was measured by reading the ELISA plate at 450 nm. The concentrations of MBP/NF-H within the samples were then determined using the standard curve. The minimum detectable amount (limit of detection, LOD) was 5 pg/ml for MBP and 0.06 ng/ml for NF-H. MBP samples above the detection range (28.4–298.4 pg/ml) were diluted (1:3); NF-H samples above the detection range (0.1–56.6 ng/ml) were diluted (1:5) and then reanalyzed with the results multiplied by the appropriate dilution factor. All samples were assayed in duplicate, and the arithmetic mean of both results was used for statistical analysis.

Furthermore, cortical and subcortical brain specimens (frontal lobes) of 10 of the cases (5 TBI cases with increasing survival times and 5 randomly chosen controls without morphological signs of traumatic injury on macroscopic or microscopic level) analyzed biochemically were collected during forensic autopsies and fixed in neutral buffered 10% formalin and then embedded in paraffin. After paraffinization, the wax blocks were sliced at 6 µm using a microtome. Consecutive sections were mounted on microscope slides and stained immunohistochemically, as previously described [42], with commercially available antibodies against MBP in a dilution of 1:40 (Diagnostic BioSystems, Pleasanton, USA), against NF-H in a dilution of 1:400 (Zytomed, Berlin, Germany), and against TMEM119 in a dilution of 1:1000 (Sigma, St. Louis, USA). Moreover, CSF cytospin preparations were stained immunocytochemically with the antibodies mentioned above using an identical dilution. The microphotographs of the brain sections and CSF cytospin preparations were taken with an Olympus DP 26 digital camera.

### Statistical analysis

Case characteristics were collected and stored with Excel Version 16.15 (Microsoft Corporation, Redmond, USA), and GraphPad Prism software version 8 was used for statistical verification (GraphPad Software, La Jolla, USA). The D’Agostino & Pearson test was used to test the parametric distribution of the samples and the sample characteristics. The biomarker levels were then analyzed using an unpaired, two-sided *t* test for normally distributed data or a Mann–Whitney *U* test for non-normally distributed data when compared to controls and between different traumatic entities. Age and PMI between the groups were compared using Mann–Whitney *U* tests. Receiver operating characteristic (ROC) curves were plotted to evaluate the area under the curve and sensitivity and specificity values of thresholds. *P* values equal to or less than 0.05 were considered statistically significant. Mean values ± standard deviations are reported in the text.

## Results

Biomarker concentrations in CSF of fatal TBI cases were compared with acute cardiac death cases as a control group. While both groups statistically differed with respect to age of the deceased (*p* < 0.05), they were matched for PMI (*p* = 0.419) and gender distribution (*p* = 0.745). The TBI cases studied here were statistically older than those of the controls.

**Fig. 2:**
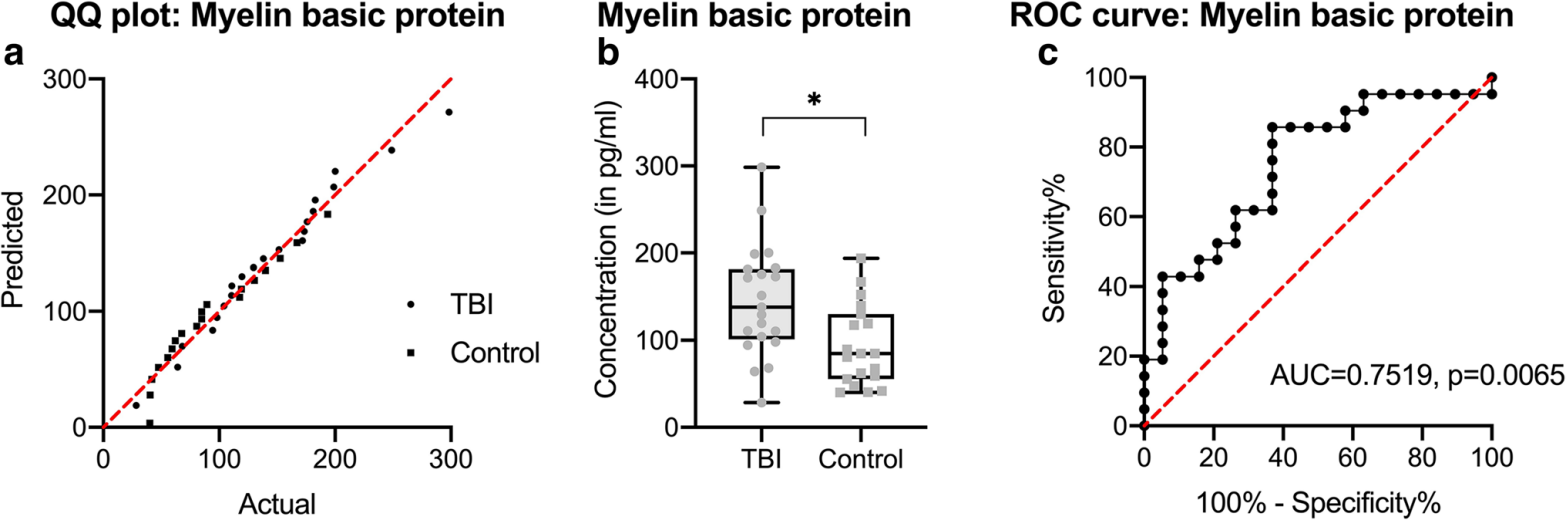
Measurement of myelin basic protein (MBP) in cerebrospinal fluid (CSF) samples of traumatic brain injury (TBI) cases and cardiovascular fatalities as controls. After testing for normality (**a**), the differences of MBP CSF levels were displayed in black-lined box plots (where the box comprises all measurements between the 25th and 75th percentile and the whiskers range from minimum to maximum) and as grayish dot plots (points for TBI, squares for controls (**b**)). CSF levels were finally tested as a receiver operating characteristics (ROC) curve for threshold determination (**c**). *p < 0.05

MBP concentrations are normally distributed within case and control groups (see Fig. 2a). TBI levels were significantly higher than in control cases (*p* = 0.006). The mean MBP concentration in CSF in the TBI group is 159.6 pg/ml, while in controls, it is 93.4 pg/ml (see Fig. 2b). A conservative threshold of > 169 pg/ml of MBP is determined with a specificity of 94.7% and a sensitivity of 42.9% (area under the curve 0.7519, see Fig. 2c). MBP CSF levels > 169 pg/ml were thus 8 times more likely to be a TBI than a cardiovascular control. There are no significant differences between TBI cases with intracranial bleedings only compared to those with additional parenchymal bleedings such as cortical contusions (*p* = 0.1346; see Supplemental Table 1).

**Fig. 3:**
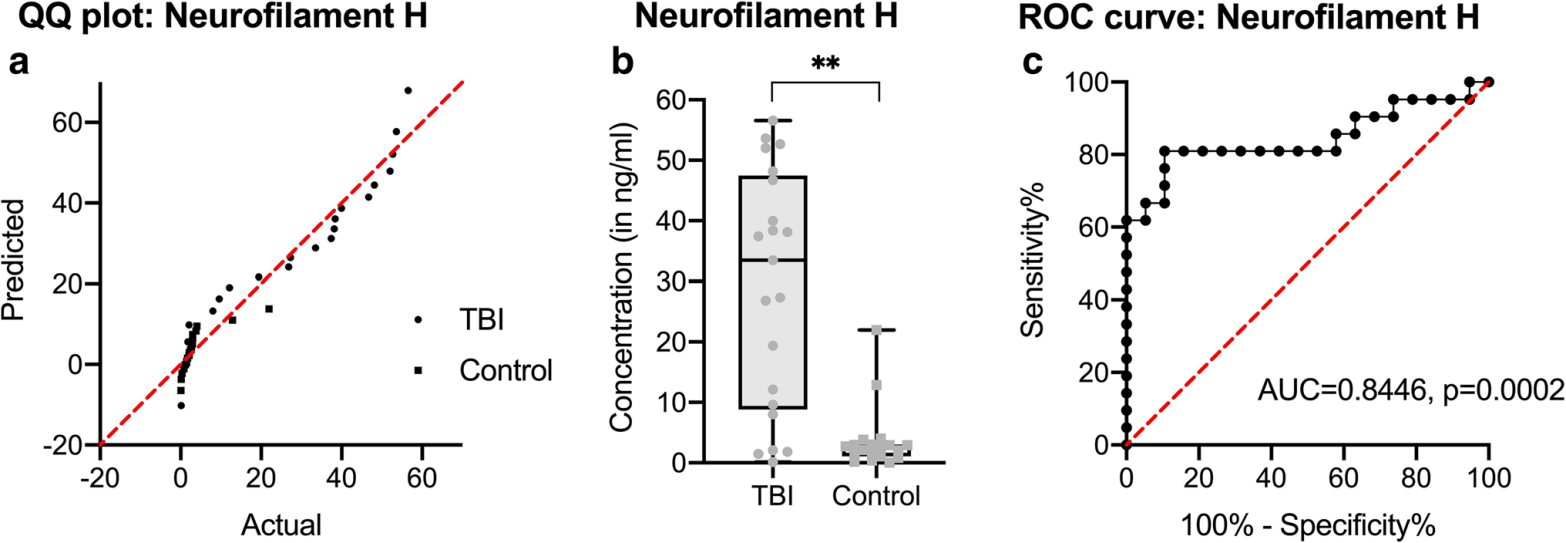
Measurement of neurofilament H (NF-H) in cerebrospinal fluid (CSF) samples of traumatic brain injury (TBI) cases and cardiovascular fatalities as controls. After testing for normality (**a**), the differences of NF-H CSF levels were displayed in black-lined box plots (where the box comprises all measurements between the 25th and 75th percentile and the whiskers range from minimum to maximum) and as grayish dot plots (points for TBI, squares for controls, see (**b**)). CSF levels were finally tested as a receiver operating characteristics (ROC) curve for threshold determination (**c**). **p < 0.001

For NF-H, there is a Gaussian distribution of readings in the case group; controls are non-parametrically distributed (see Fig. 3a). While CSF levels from control cases were largely close to LOD, the TBI group shows statistically highly significant elevated levels in CSF (*p* < 0.0001, see Fig. 3b), but no differences regarding the TBI bleeding type (*p* = 0.7240; see Supplemental Table 1). With an area under the curve of 0.8446, a conservative threshold for NF-H is found to be 6 ng/ml (specificity 98.5%, sensitivity 81%, see Fig. 3c).

**Fig. 4:**
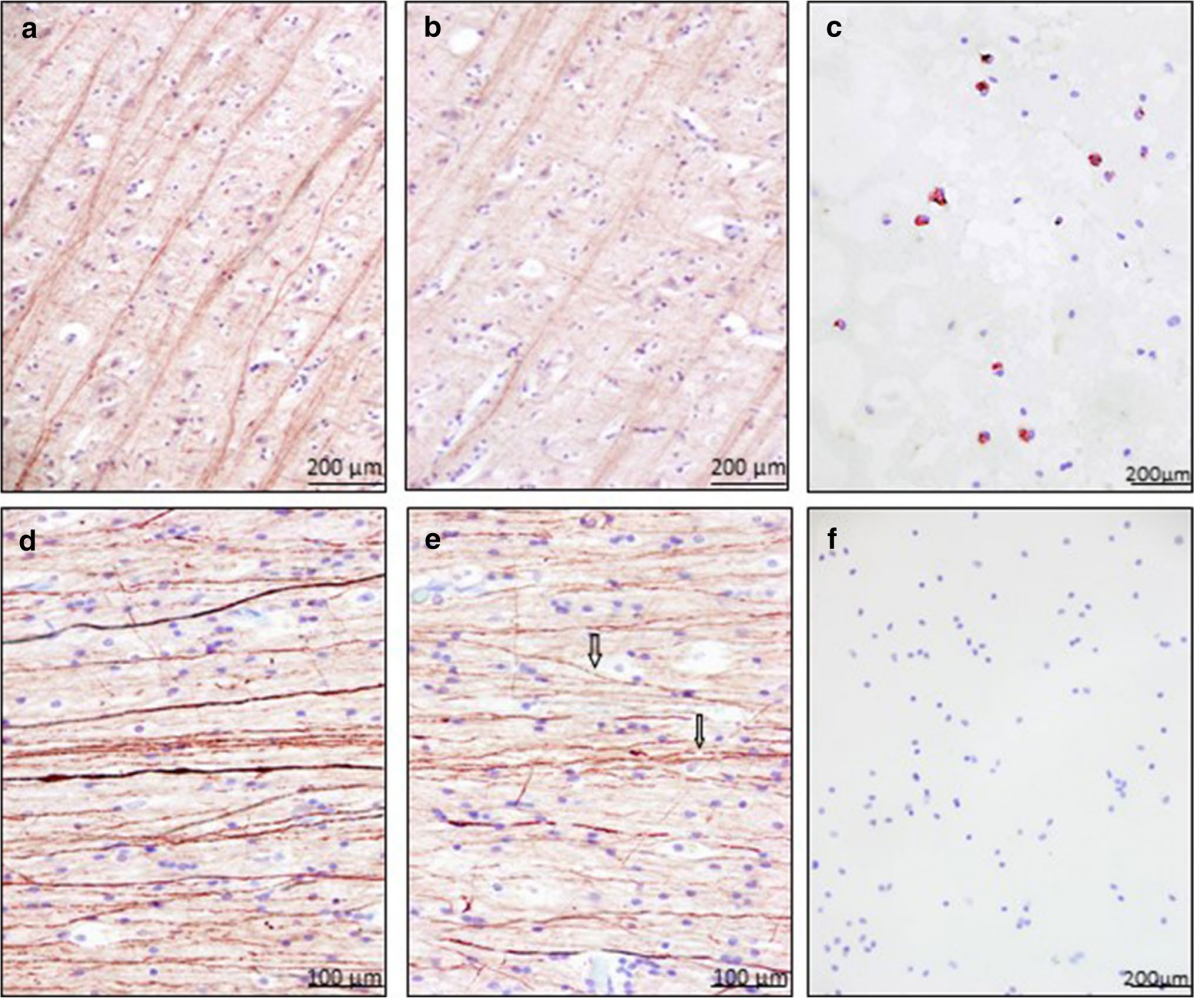
Examples of immunohistochemical staining results using anti-MBP in the cortex of a control case (**a**) and in the cortex of a traumatic brain injury (TBI) (**b**). Magnification: × 100. TBI shows a visually reduced staining reaction against MBP. NF-H in the white matter of control cases is more intensively stained (**d**) as in the white matter of TBI (**e**). Magnification: × 200. Arrows display ruptured neurofilaments. Representative immunocytochemical staining results of MBP (**c**) and NF-H (**f**) in corresponding TBI CSF samples. Whereas MBP staining in CSF was most often positive in TBI cases, NF-H staining failed to highlight this axonal biomarker in CSF via immunocytochemistry

To verify the measured levels in CSF, immunohistochemical staining against MBP as well as against NF-H was performed on randomly chosen cerebrum samples from cases also examined biochemically (5 TBI cases/5 controls). Compared with the control group (see Fig. 4a), in which there was no injury to the brain parenchyma in form of contusions or hemorrhages were identified, the TBI cases showed a visually reduced staining reaction against MBP (see Fig. 4b). Quantifications were not performed as part of this study. These stains were complemented by immunocytochemical staining of CSF against MBP. In this regard, MBP-positive phagocytic cells are detected in the CSF of four TBI cases, which showed a prolonged survival time of more than 24 h (see Fig. 4c), whereas the CSF cytochemical sections of control cases remained negative. On immunohistochemical staining against NF-H, the TBI cases showed repeatedly ruptured neurofilaments compared with the control group (see Fig. 4d + e). Immunocytochemical detection of NF-H in CSF failed despite multiple adaptations of the staining protocol (see Fig. 4f).

**Fig. 5:**
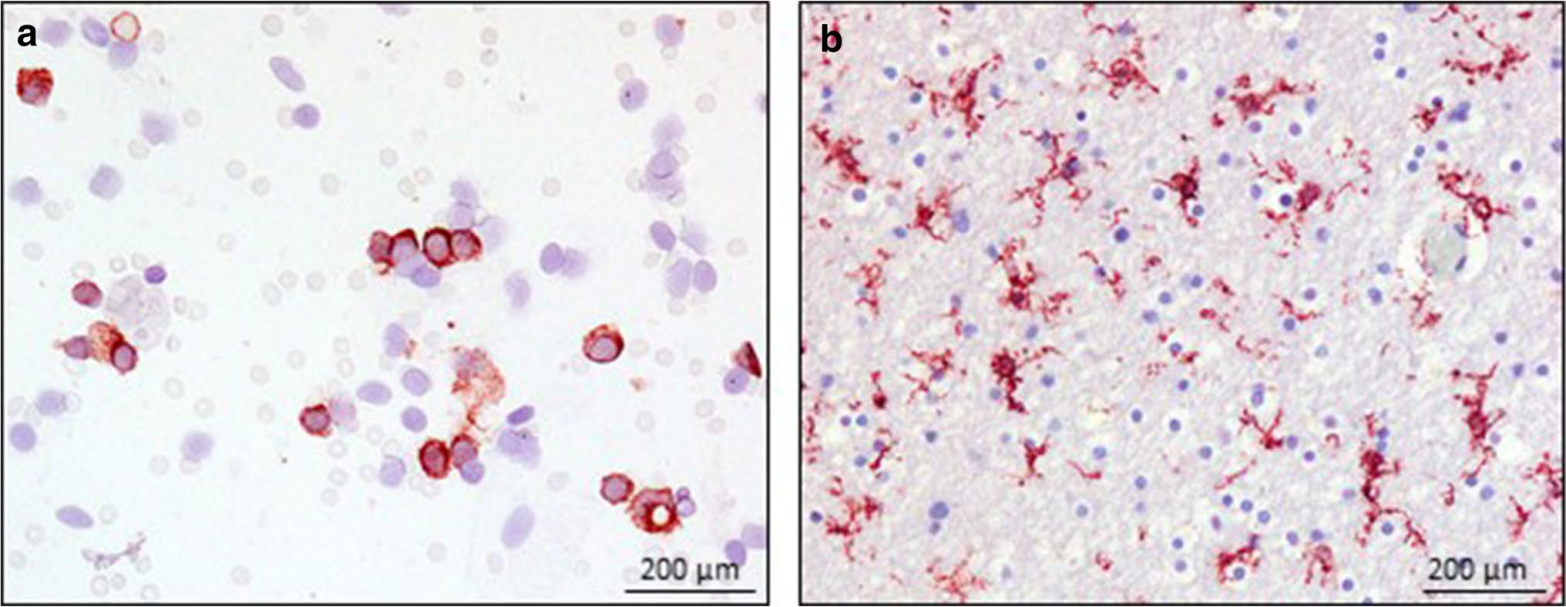
Examples of immunohistochemical and immunocytochemical staining results of TMEM119 in the CSF (**a**) and white matter (**b**) of a traumatic brain injury

When examining the response of the resident microglia and a potential interplay of microglial activation due to demyelination and axonal damage in the brain parenchyma, a marked activation of microglia in brain tissue is observed in the trauma cases (see Fig. 5b), while in the control group, predominantly ramified microglia are immunolabelled. In the CSF of the 5 TBI cases, numerous TMEM119-positive cells are detected in varying degrees of staining behavior (see Fig. 5a). Controls did not present TMEM119-positive cells in CSF.

## Discussion

In the present study focusing on biochemical determination of MBP and NF-H, both biomarkers evaluated here showed an increase in the CSF of individuals dying after TBI compared to cardiovascular controls. By determining their CSF concentration, it is thus possible to biochemically distinguish a TBI from a control group of corresponding deaths. A study by Olczak et al. [35] demonstrated the suitability of MBP alongside GFAP and NF-L as early biomarkers of lethal TBIs, although the calculated conservative threshold of MBP in that study was higher (1356.74 ± 323.66 pg/ml) than in the present paper (169 pg/ml). A possible explanation for this discrepancy in threshold determination could be that the study material of Olczak et al. included not only fatal TBI cases but also cases with minor post-traumatic neuropathological findings.

In principle, CSF, which communicates without barriers with the extracellular space of the central nervous system (CNS), i.e., with the milieu surrounding the neurons and glial cells, seems to be suitable to reflect central nervous processes such as biochemical processes in the brain after traumatization (“neuroforensomics”), not only because of its apparent postmortem stability [43]. In addition, this makes it possible to investigate CSF even in cases without visible signs of impact, and it appears promising in the light of the results presented here to identify a possible central nervous involvement and to help clarify controversial causes and circumstances of death.

Especially TBIs with a predominantly axonal component (TAI: traumatic axonal injury), clinically also referred to as DAI, may escape the expert’s attention in the (macroscopic and radiological) forensic assessment of central nervous involvement, because they leave no distinct morphological correlate in the tissue, which can be detected only by microscopical examination, for example, via the detection of “retraction bulbs and varicosities” [4, 44] or immunoreactivity to beta-amyloid precursor protein (β-APP) [45], which is said to be the gold standard for neuropathological identification of axonal injury [4, 46], but require a longer survival time of the deceased and must be distinguished from secondary hypoxic-ischemic tissue changes [47, 48].

Axonal injury can be divided into primary axotomy and secondary axotomy. Primary axotomy is a mechanical breakage of an axon resulting from forces transmitted by traumatic impact [49], whereas secondary axotomy is delayed and occurs as a result of clinical manifestations seen in DAI. Rotational acceleration of the brain can cause stretching of white matter axons, leading to a dysregulation in sodium and potassium in- and efflux, respectively, culminating in an increase in intracellular calcium concentration with pleiotropic effects within the neuron [4, 50]. One effect involves stimulation of two systems: calpain-mediated necrosis and caspase-mediated apoptosis. Calpain-mediated proteolysis predominates in the initial phase of severe TBI to result in biomarker release during this phase when sampled in human CSF [51]. Proteolytic activity results in disruption of the axonal cytoskeleton and degradation of structural proteins such as neurofilaments, MBP, Tau protein, amyloid protein, and spectrin breakdown products (SBDP) [52–54]. Due to the fact that these biomarkers are accepted to arise directly from axons, they could be a reflection of the axonal component of the TBI pathology and thus an indirect reference to TAI. There are several other biomarkers that have been studied, such as GFAP, NSE, and S-100B. While they are all of relevance to TBI, their cellular expression patterns indicate that they share no direct conceptual link with the axon itself [7, 55].

In the present paper, biochemical measurement of the axon-specific biomarkers MBP and NF-H in CSF turned out to be very suitable to distinguish TBI from a control group, reflecting the share of TAI in the lethal TBI cases studied on the basis of the immunohistochemically displayed expression patterns of both structural markers.

The detection of increased MBP und NF-H levels in CSF after TBI has to be considered carefully. Due to the fact that these biomarkers are accepted to arise directly from axons, they could be a reflection of the axonal component of TBI as well as of other forms of ischemic injury.

However, more research is needed to differentiate traumatic axonal injury from global ischemia, and further studies should include hypertensive brain hemorrhage and ischemic brain infarction data to show the effects of hypoxia of brain tissue without traumatic impact on CSF biomarker levels.

Since the orchestration of neuroinflammation after TBI is multiform and complex [56], a multi-methodological approach was used in the present study in addition to the primarily biochemical investigation of CSF, viz., immunohistochemistry of traumatized brain tissues or immunocytochemistry of CSF, to additively confirm the biochemical evidence of a potential traumatization of the brain parenchyma. Elevated MBP and NF-H levels in CSF were associated with a reduced staining response of the myelin sheath in demyelination and the presence of ruptured neurofilaments, respectively [35], whereas control cases with low MBP and NF-H CSF levels did not show comparable immunohistochemical changes in the white matter.

The observation of increased MBP and NF-H levels of CSF after TBI was further supported by the fact that concomitant activation of microglia could be demonstrated in the respective brain tissue, but in the control cases, and thus with low values of MBP and NF-H, more ramified microglia were detected in CSF. As already mentioned in previous publications, a special role as a stimulus of the resident microglia was attributed to the released myelin after damage of the myelin sheath with subsequent activation [57, 58]. In addition to a very early response of microglia after TBI, one of our own publications also showed activation of the so-called M2 microglia/macrophages, which contribute to the regeneration of injured brain tissue through their phagocytic activity [19]. Demyelination, such as after TBI, results in an increased release of lipid components such as phospholipids and cholesterol, which are major constituents of myelin and are phagocytosed. This may explain the presence of “fat-containing” macrophages [59]. Up to now, the literature contains little information as to when these “fat-containing” macrophages begin to appear [60]. According to Oehmichen et al., they were detected after 17 h, after 5–6 days, and in one case even 30 years after, a TBI had occurred [59]. Thus, the detection of “fat-containing” macrophages in the tissue may support the observation in TBIs with a prolonged survival time to find MBP-positive macrophages in CSF, as also illustrated here. Moreover, their detection in the CSF seems to be not only due to a longer survival time but also to a scenario of a “lagging behind” of the CSF, as a result of a so-called passage delay, to the brain parenchyma, which could be repeatedly observed in our cases, since phagocytosis primarily takes place in the tissue, i.e., at the site of direct trauma, and the phagocytically active cells can only afterwards enter the CSF. Thus, the time passing before entry into the extracellular space could play a role before CSF immunocytochemistry will return positive for here discussed axonal biomarkers. This potential time latency is currently being investigated in an accompanying study and will be reported separately.

NF-H could not be detected by immunocytochemical staining of CSF. A possible explanation for this could be that in the rat brain, a postmortem accumulation of neurofilaments takes place in the perikaryon [61]. In addition, other studies reported that NF-H is particularly stable compared with NF-M/L because it has the highest degree of phosphorylation (dephosphorylation increases sensitivity to enzymatic degradation) and the ability to bind to calmodulin [62, 63]. For this reason, it seems hypothetically conceivable that in the course of traumatization and the associated damage to the axonal cytoskeleton, there is a temporary storage of NF-H and other neurofilaments in the perikaryon, which, however, is interrupted by the progressive neuronal damage, thus leading to the fact that the neurofilaments, bypassing phagocytosis or consciously using other transport mechanisms, which requires a living organism (vital reaction), reach the extracellular space, where they can be detected biochemically in CSF, but remain masked for methods like immunocytochemistry.

## Limitations

In our study, we used, on the one hand, a heterogeneous study material with a large and statistically divergent age range and different postmortem intervals, but representing our daily autopsy material. On the other hand, factors such as the ambient temperature of the body at the time of death, freezing of CSF samples for storage until measurement, as well as undetected neurodegenerative diseases or past minor traumas, may influence the concentration levels of the measured biomarkers. We tried to rule this out by strict sample selection with exclusion of chronic neurodegenerative diseases and repetitive trauma. To establish the relationship between postmortem MBP und NF-H levels and brain tissue damage as sign of TBI including axonal components, further investigations are necessary to differentiate between direct traumatic axonal damage and secondary ischemic injury.

Attempts were made to cohort different survival times in the TBI group in order to arrive at a conclusion about basic post-traumatic changes. A comparison of the measured values with each other with regard to the length of survival time has not been performed due to the small number of cases.

At the beginning of the present study, a control group of cardiovascular fatalities was defined to allow a representative comparison of one of the most common causes of (natural) death in the forensic autopsy material with TBI cases. However, this preselection does not allow to compare the results uncritically with other causes of death, such as hypoxia following strangulation or cerebral hemorrhage from an internal cause. Additionally, these control cases chosen might be influenced by heterogeneous effects such as different times of agony. In particular, the cases of acute myocardial infarction could present a long(er) period of agony which could play a role in secondary CNS ischemia.

The definition of a control group was necessary to keep the study within an economically reasonable scope. The immunohistochemical and immunocytochemical examinations were also only possible on a representative basis due to budget constraints, and further studies should include immunoreactivity of β-APP for the aforementioned reasons.

## Conclusion

In conclusion, the present study focusing on postmortem biochemical analysis demonstrated that MBP and NF-H are promising cerebral neuroinjury biomarkers that appear suitable to differentiate TBI from cardiovascular death. The multi-methodological approach via immunohistochemical and immunocytochemical staining can help to verify biochemical results and supplies an additional tool in the forensic neuropathological interpretation of TBIs.

## Supplementary Information

Below is the link to the electronic supplementary material.

Supplementary file1 (DOCX 20 KB)
