# Supplementary material for: Myelin basic protein and neurofilament H in postmortem cerebrospinal fluid as surrogate markers of fatal traumatic brain injury
Source: Int J Legal Med. 2021 Apr 24;135(4):1525–35. doi: 10.1007/s00414-021-02606-y (PMC8205912; doi:10.1007/s00414-021-02606-y)
Supplement: Supplementary file 1 — Supplementary file1 (DOCX 20 KB) [file 414_2021_2606_MOESM1_ESM.docx]

| **Case**  **number** | **Intracranial hemorrhage [MBP]**  **pg/ml** | **Cortical contusion [MBP]**  **pg/ml** |
| --- | --- | --- |
|  |  |  |
| 1 | 171.9 | 104.0 |
| 2 | 182.8 | 198.8 |
| 3 | 138.2 | 298.3 |
| 4 | 181.1 | 98.2 |
| 5 | 94.4 | 129.5 |
| 6 | 173.3 | 151.5 |
| 7 | 200.1 | 248.9 |
| 8 | 110.6 | 175.9 |
| 9 | 64.2 | 119.5 |
| 10 | 68.2 |  |
| 11 | 28.4 |  |
| 12 | 110.9 |  |

| **Case**  **number** | **Intracranial hemorrhage [NF-H]**  **ng/ml** | **Cortical contusion**  **[NF-H]**  **ng/m** |
| --- | --- | --- |
|  |  |  |
| 1 | 27.3 | 8.0 |
| 2 | 56.5 | 48.2 |
| 3 | 1.8 | 1.5 |
| 4 | 53.6 | 26.8 |
| 5 | 12.1 | 33.5 |
| 6 | 19.3 | 37.4 |
| 7 | 38.1 | 38.4 |
| 8 | 46.7 | 39.9 |
| 9 | 2.1 | 9.6 |
| 10 | 52.6 |  |
| 11 | 0.1 |  |
| 12 | 52.0 |  |

**Supplemental Table 1:** Cerebrospinal fluid (CSF) levels of myelin basic protein (MBP) and neurofilament H (NF-H) differentiated in subgroups of traumatic brain injury; intracranial hemorrhages (epidural, subdural, subarachnoid hemorrhage) and intracerebral hemorrhages such as cortical contusions. No significant differences were calculated for both biomarkers.
